# Determinants of abnormal liver-related biomarkers in adult COVID-19 patients

**DOI:** 10.3389/fcimb.2025.1662585

**Published:** 2026-01-07

**Authors:** Sijia Zhang, Yue Hu, Li Liu, Xianying Ning, Qiang Li, Guangqin Xiao

**Affiliations:** 1 Cancer Center, Union Hospital, Tongji Medical College, Huazhong University of Science and Technology, Wuhan, China; 2 Institute of Radiation Oncology, Union Hospital, Tongji Medical College, Huazhong University of Science and Technology, Wuhan, China; 3 Hubei Key Laboratory of Precision Radiation Oncology, Wuhan, China; 4 Department of Biophysics, Center for Integrative Physiology and Molecular Medicine (CIPMM), School of Medicine, Saarland University, Homburg, Germany; 5 Department of Biomedical Sciences, Institute for Health Research and Education, Osnabrück University, Osnabrück, Germany; 6 Department of Epidemiology, Harvard T.H. Chan School of Public Health, Boston, MA, United States; 7 Clinical and Translational Epidemiology Unit, Massachusetts General Hospital and Harvard Medical School, Boston, MA, United States

**Keywords:** liver injury, COVID-19, abnormal hepatic injury markers, normal hepatic injury markers, adult patients

## Abstract

**Background:**

Serum hepatic injury markers indexes are altered in COVID-19 patients. We aimed to explore the factors that could be associated with abnormal serum hepatic injury markers in adult COVID-19 patients.

**Methods:**

Eight main hepatic injury markers were examined. Demographic and hematological information, mean CT values (MCTVs) of liver and pancreas, and abdominal subcutaneous fat thickness were recorded. Regression analysis was conducted to identify factors related to abnormal hepatic injury markers.

**Results:**

1,007 adult COVID-19 patients (444 males and 563 females) were included, among whom 697 patients (69.2%) had at least one abnormal hepatic injury markers marker. Females had lower risks of elevated Total Bilirubin (TBil), Direct Bilirubin (Dbil), ALT, AST, GGT and decreased albumin, with ORs of 0.61 (95%CI: 0.42-0.89), 0.36 (95%CI: 0.16-0.83), 0.20 (95%CI: 0.12-0.32), 0.42 (95%CI: 0.30-0.58), 0.36 (95%CI: 0.22-0.60) and 0.40 (95%CI: 0.30-0.54). Patients with greater ratios of subcutaneous fat thickness to abdominal diameters also had lower risks of abnormalities in these six markers. Older patients had higher serum levels of AST but lower levels of albumin and ALT. The risks of abnormal DBil and AST were 3.26 and 1.62 times higher in patients with a history of HBV infection. Patients with many abnormal hepatic injury markers indexes had significantly lower MCTVs of liver and pancreas and higher levels of fibrinogen and LDH in blood.

**Conclusions:**

Sex, age, HBV infection, fibrinogen, LDH, liver and pancreas MCTVs, and ratio of abdominal subcutaneous fat thickness to the sum of the abdominal diameters were independently associated with many abnormal serum hepatic injury markers in adult COVID-19 patients.

## 1 Background

Since cases of coronavirus disease 2019 (COVID-19) were first observed in 2019, the disease has spread globally (Gudbjartsson et al., 2020; Muttineni et al., 2021; Weinreich et al., 2021). To date, there have been more than 120 million COVID-19 cases, and over 2.6 million people have died of the disease worldwide (Tanne, 2020; Williamson et al., 2020; Marofi et al., 2021). The disease, caused by SARS-CoV-2, has created a major public health event threatening human life and health. In addition to causing lung damage, SARS-CoV-2 can invade multiple organs in humans, resulting in organ injury (Ackermann et al., 2020; Young et al., 2020). Nie and colleagues found that key factors associated with hypoxia, angiogenesis, blood coagulation, and fibrosis in multiple organs are dysregulated in COVID-19 patients (Nie et al., 2021). Shen’s research has revealed that protein and metabolite changes occur in multiple organs in COVID-19 patients (Shen et al., 2020). Among the human organs attacked by SARS-CoV-2, the liver is especially vulnerable to virus-induced injury (Higuera-de la Tijera et al., 2021; Marjot et al., 2021). Studies have shown that liver dysfunction is closely related to hospital admission and mortality among COVID-19 patients (Li et al., 2020; Weber et al., 2021).

The liver is an important organ for metabolism and detoxification (Fuchs et al., 2017; Massafra et al., 2017; Berndt et al., 2021). SARS-CoV-2 can impair hepatocytes directly or indirectly via toxins, which can lead to liver injury and cause acute diffuse liver diseases (Li et al., 2020; Hofman et al., 2021; Nie et al., 2021; Weber et al., 2021). The proportion of COVID-19 patients with abnormal hepatic injury markers is extremely high (Dahiya et al., 2020; Marjot et al., 2021; Weber et al., 2021). However, some studies have focused only on a few common indicators of hepatic injury markers, such as bilirubin, albumin, alanine aminotransferase (ALT) and aspartate aminotransferase (AST). In addition to these common indicators, other indicators, such as alkaline phosphatase (ALP), gamma-glutamyl transferase (GGT), and total bile acid (TBA) in serum, can also reflect hepatic injury markers in humans. Exploration of the association of COVID-19 with abnormalities in only one or two serum hepatic injury markers indexes may bias the results (Roedl et al., 2021). The proportions of COVID-19 cases with abnormal hepatic injury markers have differed among various studies (Li et al., 2020; Pang et al., 2020; Marjot et al., 2021; Weber et al., 2021). In addition, the percentages of COVID-19 patients who have one, two or more abnormal hepatic injury markers indicators simultaneously are unknown. Some patients with SARS-CoV-2 infection have normal serum hepatic injury markers indexes, and the differences between COVID-19 patients with abnormal hepatic injury markers indexes and those with normal hepatic injury markers indexes are unclear. Moreover, whether the abnormal hepatic injury markers in COVID-19 patients is related to factors other than liver damage caused by SARS-CoV-2 is ambiguous.

In this study, we focused on eight main serum hepatic injury markers in adult COVID-19 patients. We analyzed demographic data, blood test results and indicators on unenhanced CT scans in patients with abnormal or normal hepatic injury markers. We aimed to identify the potential factors associated with abnormalities in each hepatic injury markers marker in adult COVID-19 patients.

## 2 Methods

We retrospectively collected data for COVID-19 patients hospitalized in Wuhan Union Hospital from February 2020 to December 2022. A COVID-19 diagnosis is defined as a laboratory-confirmed infection with the SARS-CoV-2 virus, which is the virus that causes the disease COVID-19. This is usually done through a PCR test, which detects the genetic material of the virus. The general demographic characteristics and medical histories of the patients were recorded. We collected the hematological indexes of the COVID-19 patients within one week of hospitalization. Data on blood cell counts, immune cell percentages, indicators of coagulatory function, serum inflammatory indicators, hepatic injury markers, renal function indicators and other serum biochemical indexes were also collected. Patients younger than 18 year and patients without serological liver marker data were excluded.

Patients received unenhanced CT scans within one week after admission. The instrument was a Siemens SOMATOM Definition 64-slice spiral CT scanner. The CT data were based on the picture archiving and communication system (PACS) using the standard compression algorithm supported by DICOM 3.0. CT data collection was carried out by two researchers (XGQ and NXY) who were trained by a senior radiologist, and they were blinded to the patient information. Discrepancies in results between the two researchers were resolved by calculating the average values. We measured the mean CT value (MCTV) of the liver, the MCTV of the spleen, the MCTV of the pancreas, the abdominal subcutaneous fat thickness, abdominal transverse and anteroposterior diameters in the plane of the superior mesenteric artery originating from the abdominal aorta (**Supplementary Figure 1**) and other indicators for each individual using PACS software. We further assessed model performance to ensure robustness. Multicollinearity among independent variables was examined using the variance inflation factor (VIF), and no significant collinearity was detected. Model adequacy and goodness-of-fit were verified using the Hosmer–Lemeshow test, confirming satisfactory model stability.

IBM SPSS software (Version 22.0) was used to conduct data analysis. Independent t-tests and chi square tests were conducted to compare the variables between the normal and abnormal hepatic injury markers groups. All variables with *P* values less than 0.2 were selected for binary logistic regression analysis to identify potential factors associated with each abnormal serum hepatic injury markers marker. A *P* value less than 0.05 was considered to indicate statistical significance.

## 3 Results

**Figure 1:**
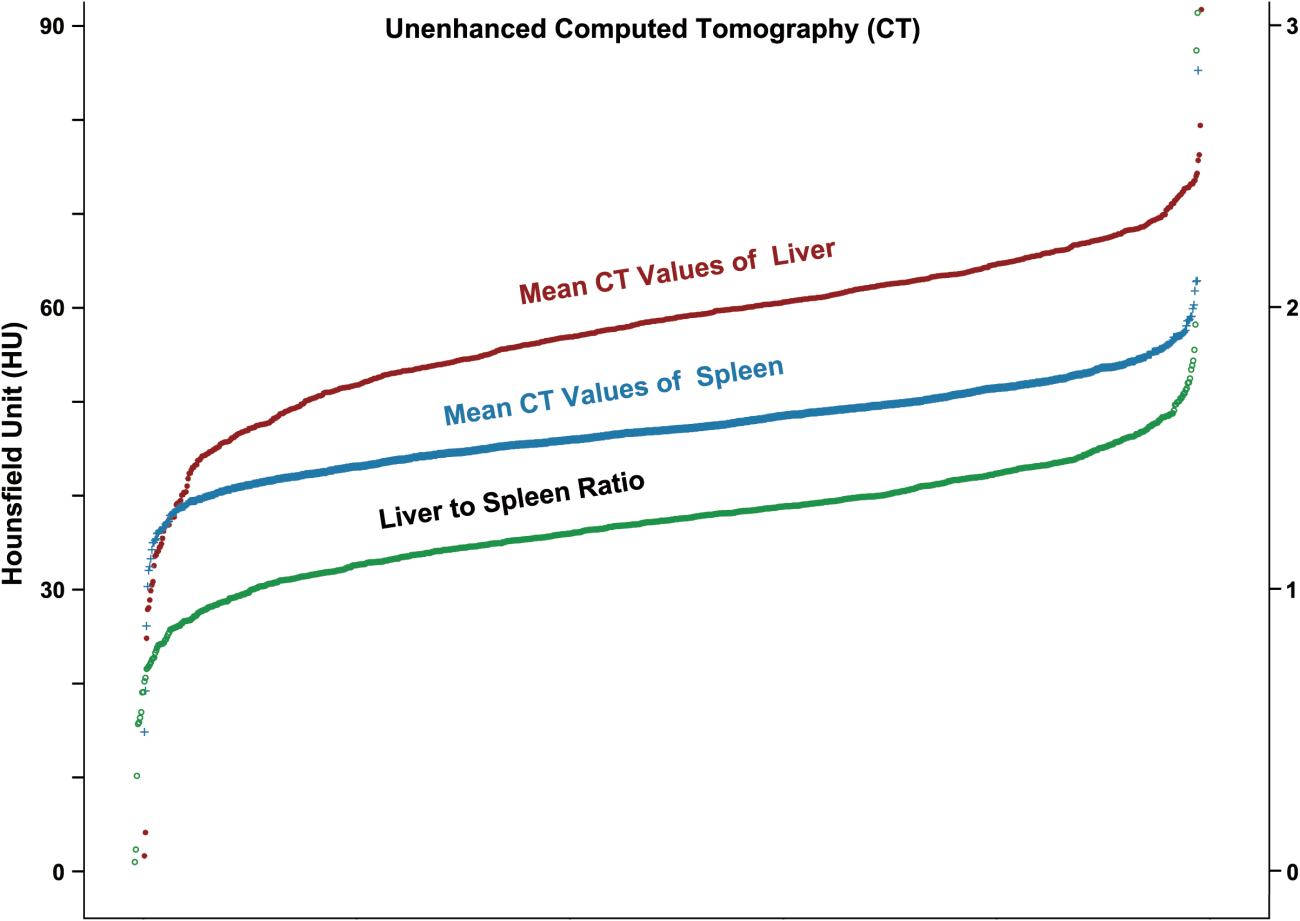
Scatter plot of the liver MCTVs, spleen MCTVs and liver MCTV/spleen MCTV ratios of adult COVID-19 patients.

Ten patients were excluded from this study (1 was younger than 18 y, and 9 lacked hepatic injury markers results). A total of 1,007 adult COVID-19 patients were included. There were 444 males (44.1%) and 563 females (55.9%). The mean age of all patients was 59.9 y. Among the patients, 129 had a history of hepatitis B virus (HBV) infection. Unenhanced CT was performed on 983 patients (97.6%). The mean liver MCTV was 57.7 HU. Thirty-five patients (3.6%) had liver MCTVs less than 40 HU, and 33 (3.4%) had liver MCTVs greater than 70 HU. There were 109 patients (11.1%) with a liver MCTV/spleen MCTV ratio less than 1.0 and 93 (9.5%) with a ratio greater than 1.5 (**Figure 1**, **Table 1**).

**Figure 2:**
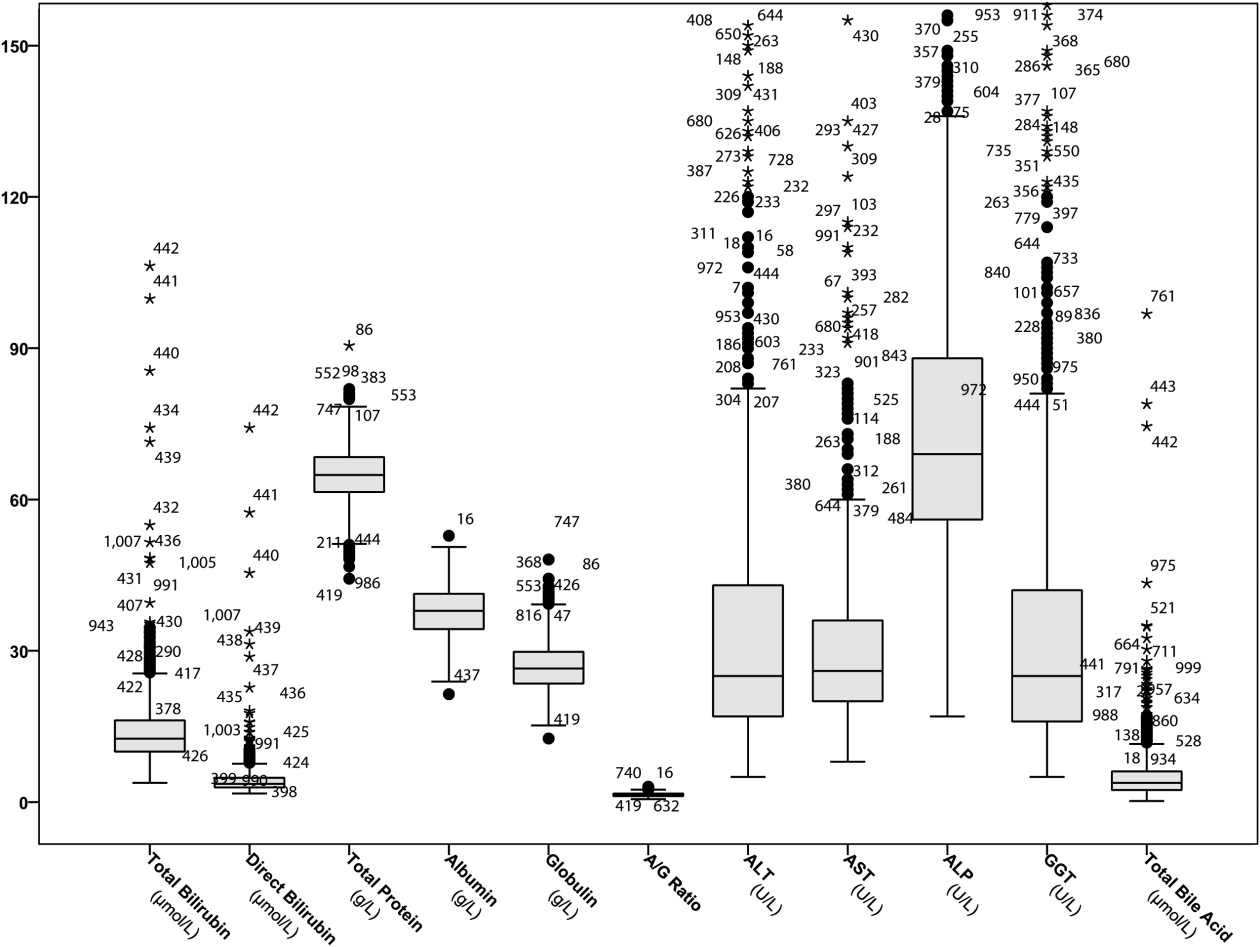
Boxplot of serum indicators of hepatic injury markers in adult COVID-19 patients.

**Figure 3:**
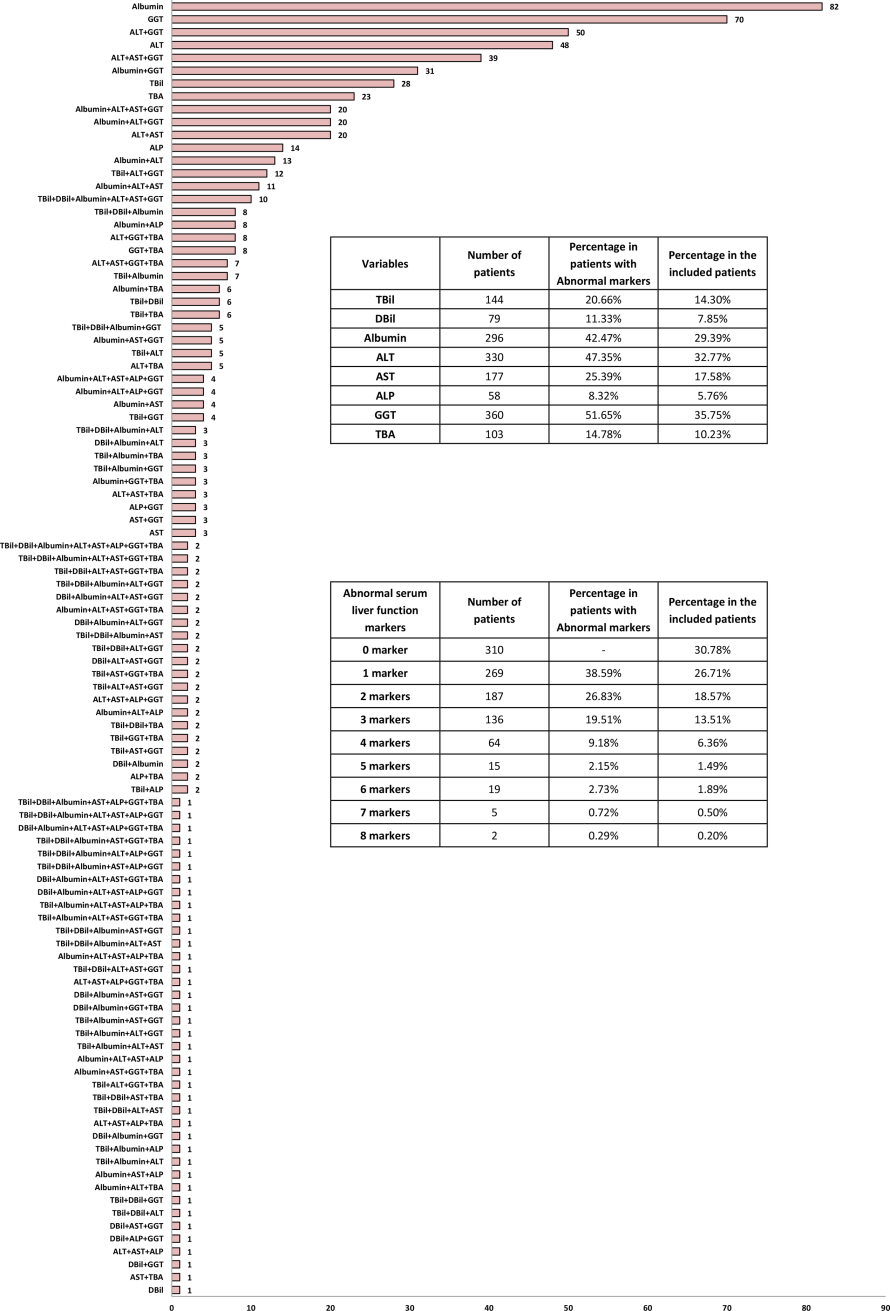
Distribution of the numbers of adult COVID-19 patients with abnormalities in different serum hepatic injury markers.

**Figure 4:**
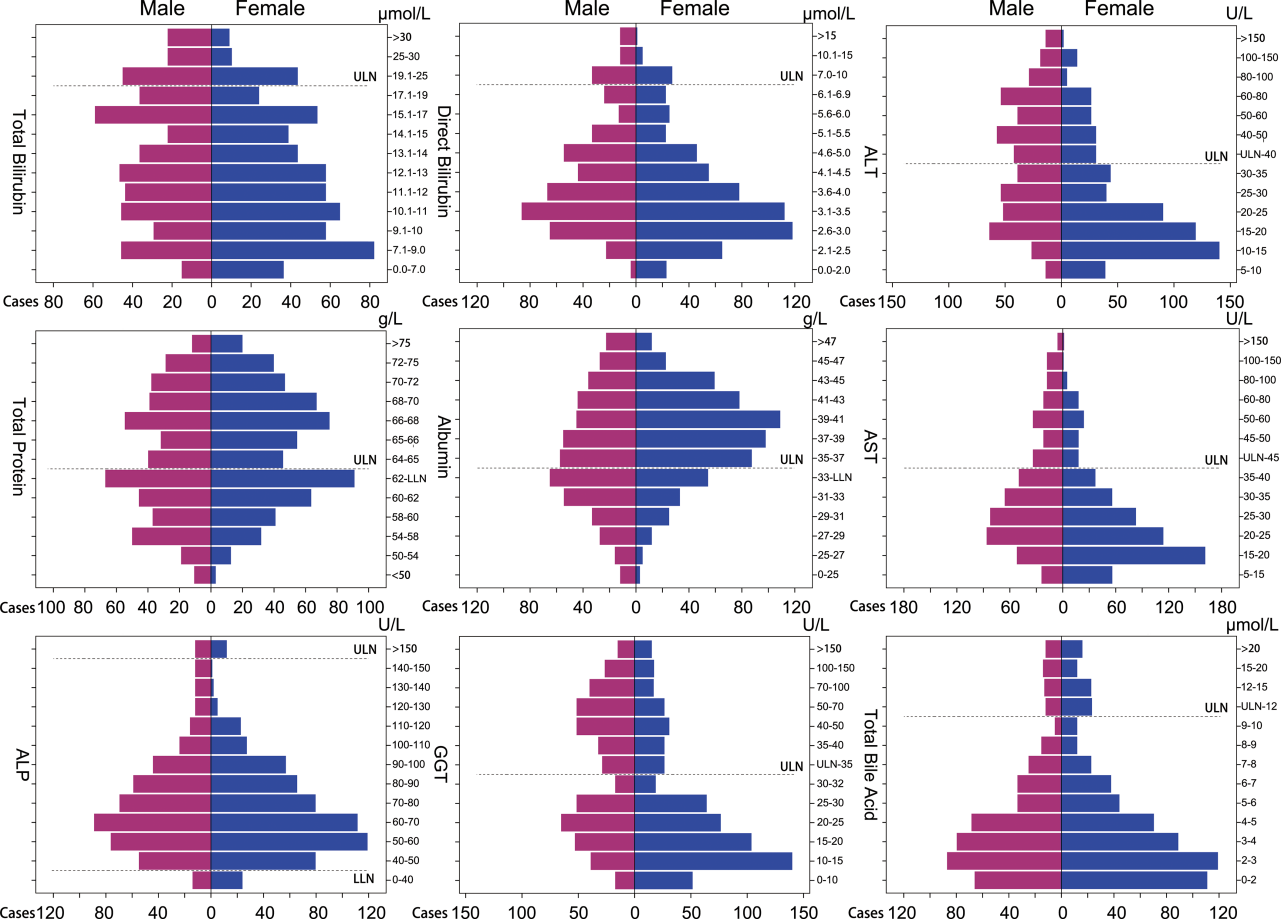
Population pyramids of serum hepatic injury markers indexes in adult COVID-19 patients.

**Figure 5:**
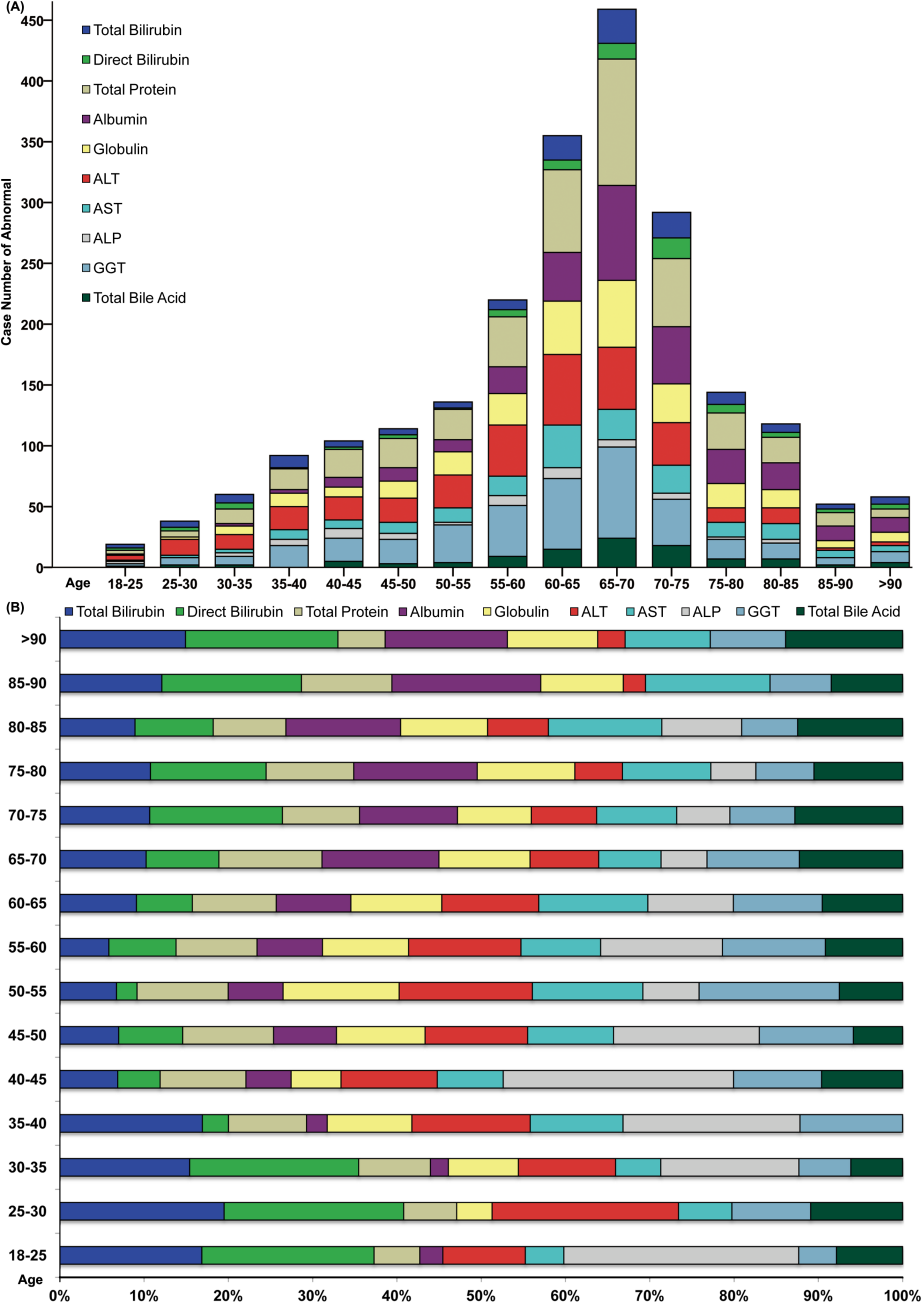
Case **(A)** and percentage **(B)** distributions of adult COVID-19 patients with abnormal serum hepatic injury markers indexes.

**Figure 2** displays the distribution of values for each serum hepatic injury markers index among all patients. A total of 697 patients (69.2%) had at least one abnormal serum hepatic injury markers marker. There were 269 patients (26.7%) with only one abnormal marker, 187 patients (18.6%) with two, 136 patients (13.5%) with three, 64 patients (6.4%) with four, 15 patients (1.5%) with five, 19 patients (1.9%) with six, 5 patients (0.5%) with seven, and 2 patients (0.2%) with eight abnormal hepatic injury markers indexes. **Figure 3** shows the number of patients with abnormal values for each serum hepatic injury markers marker. The distributions of male and female COVID-19 patients with different levels of serum hepatic injury markers indicators are presented in **Figure 4**. **Figure 5** displays the numbers and percentages of patients with various abnormal hepatic injury markers indexes grouped by age.

One hundred forty-four patients (14.3%) had abnormal serum total bilirubin (TBil). The proportion of males (82/362) with elevated TBil was higher than that of females (62/501) *(P = 0.001)*. Among patients less than sixty years old, 11.6% (48/414) had elevated TBil; among patients more than sixty years old, 16.2% (96/593) had elevated TBil. **Table 2** shows that the values of 17 blood variables were significantly different between the abnormal and normal TBil groups. **Table 3** demonstrates that the mean liver MCTV of patients with elevated TBil (55.9 HU) was lower than that of patients with normal TBil (58.0 HU) *(P = 0.006)*. The patients with elevated TBil had thinner abdominal subcutaneous fat layers and lower ratios of abdominal subcutaneous fat thickness to thoracic or abdominal diameters, with all *P* values less than 0.05 (**Table 3**). **Table 1** shows the factors associated with elevated serum TBil, which included sex (odds ratio, OR: 0.61, 95% confidence interval, CI: 0.42-0.89), smoking (OR: 2.43, 95% CI: 1.09-5.42), liver MCTV (OR: 0.13, 95% CI: 0.03-0.58) and the ratio of abdominal subcutaneous fat thickness to the sum of abdominal anteroposterior and transverse diameters (OR: 0.06, 95% CI: 0.01-0.36).

**Table 1:** Comparison of the demographic characteristics of adult COVID-19 patients between the abnormal and normal serum hepatic injury markers index groups.

Variables		Total Bilirubin			Direct Bilirubin			Albumin			ALT			AST			ALP					GGT			Total Bile Acid		
		>ULN (n=144)	Normal (n=863)	P	>ULN (n=79)	Normal (n=928)	P	<LLN (n=296)	Normal (n=711)	P	>ULN (n=330)	Normal (n=677)	P	>ULN (n=177)	Normal (n=830)	P	<LLN (n=38)	>ULN (n=20)	Normal (n=949)	P (L)	P (U)	>ULN (n=360)	Normal (n=647)	P	>ULN (n=103)	Normal (n=904)	P
**Gender**	Male	82	362	0.001	52	392	<0.001	177	267	<0.001	220	224	<0.001	110	334	<0.001	14	12	418	0.38	0.155	225	219	<0.001	49	395	0.453
	Female	62	501		27	536		119	444		110	453		67	496		24	8	531			135	428		54	509	
**Mean Age (y)**		61.6	59.7	0.192	64.0	59.6	0.011	68.2	56.5	<0.001	58.0	60.9	0.004	63.7	59.2	<0.001	53.7	59.1	60.2	0.008	0.733	61.1	59.3	0.061	65.3	59.3	<0.001
**Age Groups-y**	18-50	35	211	0.004	16	230	<0.001	25	221	<0.001	87	159	0.029	30	216	0.001	18	5	223	0.022	0.706	72	174	0.063	13	233	0.001
	50-60	13	155		7	161		32	136		69	99		28	140		5	5	158			73	95		13	155	
	60-70	48	320		21	347		118	250		109	259		60	308		10	5	353			133	235		39	329	
	70-80	31	117		24	124		75	73		47	101		35	113		3	4	141			54	94		25	123	
	>80	17	60		11	66		46	31		18	59		24	53		2	1	74			28	49		13	64	
**Weight (kg)**		63.1	62.3	0.687	64.5	62.2	0.277	62.9	62.3	0.674	65.9	60.6	<0.001	62.6	62.4	0.936	59.4	61.2	62.6	0.35	0.729	66.2	60.8	<0.001	59.9	62.7	0.206
**Height (cm)**		164.8	163.5	0.223	166.2	163.4	0.034	164.1	163.5	0.459	166.2	162.4	<0.001	164.7	163.5	0.232	162.8	162.8	163.7	0.633	0.687	165.5	162.9	0.002	164.2	163.6	0.675
**Mean BMI**		23.2	23.2	0.992	23.3	23.2	0.881	23.3	23.2	0.841	23.7	22.9	0.041	22.9	23.3	0.432	22.4	23.0	23.3	0.391	0.839	24.1	22.8	0.001	22.1	23.3	0.072
**BMI Groups**	<18	3	9	0.407	3	9	0.121	4	8	0.914	1	11	0.071	2	10	0.904	0	0	12	0.444	0.549	1	11	0.002	2	10	0.316
	18-24	26	193		16	203		58	161		71	148		36	183		10	5	204			55	164		20	199	
	14-28	16	78		10	84		27	67		38	56		13	81		2	4	88			42	52		8	86	
	>28	3	23		4	22		8	18		12	14		5	21		0	0	26			10	16		0	26	
**Hypertension**	No	74	489	0.238	39	524	0.223	145	418	0.004	176	387	0.25	90	473	0.135	21	13	529	0.953	0.409	175	388	0.001	50	513	0.112
	Yes	70	374		40	404		151	293		154	290		87	357		17	7	420			185	259		53	391	
**Coronary Atherosclerosis**	No	130	805	0.196	69	866	0.048	264	671	0.004	301	634	0.159	155	780	0.003	36	19	880	0.639	0.698	331	604	0.405	96	839	0.883
	Yes	14	58		10	62		32	40		29	43		22	50		2	1	69			29	43		7	65	
**Type 2 Diabetes**	No	92	606	0.127	44	654	0.006	188	510	0.01	213	485	0.022	106	592	0.003	31	10	657	0.104	0.066	214	484	<0.001	60	638	0.01
	Yes	52	257		35	274		108	201		117	192		71	238		7	10	292			146	163		43	266	
**Operation History**	No	111	594	0.045	63	642	0.049	210	495	0.676	239	466	0.243	130	575	0.272	27	13	665	0.897	0.624	251	454	0.882	76	629	0.377
	Yes	33	269		16	286		86	216		91	211		47	255		11	7	284			109	193		27	275	
**Allergic History**	No	128	783	0.486	74	837	0.312	276	635	0.053	302	609	0.429	164	747	0.275	36	19	856	0.352	0.473	326	585	0.943	94	817	0.772
	Yes	16	80		5	91		20	76		28	68		13	83		2	1	93			34	62		9	87	
**HBV Infection**	No	124	754	0.676	65	813	0.174	252	626	0.208	283	595	0.342	145	733	0.021	30	16	832	0.113	0.304	310	568	0.445	84	794	0.071
	Yes	20	109		14	115		44	85		47	82		32	97		8	4	117			50	79		19	110	
**Smoking**	No	134	841	0.005	74	901	0.096	278	697	0.001	310	665	<0.001	171	804	0.859	38	19	918	0.258	0.668	337	638	<0.001	100	875	0.871
	Yes	10	22		5	27		18	14		20	12		6	26		0	1	31			23	9		3	29	
**Alcohol Drinking**	No	139	850	0.099	76	913	0.16	285	704	0.003	323	666	0.577	174	815	0.918	38	19	932	0.405	0.293	347	642	0.001	101	888	0.901
	Yes	5	13		3	15		11	7		7	11		3	15		0	1	17			13	5		2	16	

* ULN, Upper Limit of Normal; LLN, Lower Limit of Normal; ALT, Alanine Aminotransferase; AST, Aspartate Aminotransferase; ALP, Alkaline Phosphatase; GGT, Gamma-glutamyl Transferase; BMI, Body Mass Index; HBV, Hepatitis B Virus.

**Table 2:** Comparison of the laboratory test results of adult COVID-19 patients between the abnormal and normal serum hepatic injury markers index groups.

Variables	Total Bilirubin			Direct Bilirubin			Albumin			ALT			AST			ALP					GGT			Total Bile Acid		
	>ULN (n=144)	Normal (n=863)	P	>ULN (n=79)	Normal (n=928)	P	<LLN (n=296)	Normal (n=711)	P	>ULN (n=330)	Normal (n=677)	P	>ULN (n=177)	Normal (n=830)	P	<LLN (n=38)	>ULN (n=20)	Normal (n=949)	P (L)	P (U)	>ULN (n=360)	Normal (n=647)	P	>ULN (n=103)	Normal (n=904)	P
**WBC (10^9/L)**	6.8	5.7	<0.001	7.6	5.7	<0.001	6.6	5.5	<0.001	6.2	5.6	<0.001	6.5	5.7	0.009	4.9	7.4	5.8	0.024	0.176	6.3	5.5	<0.001	5.9	5.8	0.824
**RBC (10^12/L)**	3.9	3.9	0.644	3.8	3.9	0.300	3.7	4.0	<0.001	4.0	3.8	<0.001	3.9	3.9	0.679	3.7	3.5	3.9	0.022	0.071	3.9	3.9	0.897	3.8	3.9	0.042
**Hemoglobin (g/L)**	123.5	122.8	0.702	120.4	123.1	0.257	116.3	125.7	<0.001	128.0	120.4	<0.001	124.1	122.7	0.311	117.4	112.0	123.4	0.003	0.083	123.9	122.4	0.162	121.6	123.1	0.375
**Platelet (10^9/L)**	202.8	236.0	<0.001	203.6	233.6	0.004	241.8	226.9	0.024	245.6	224.3	0.001	235.1	230.4	0.585	231.4	244.8	231.0	0.977	0.674	243.7	224.4	0.002	212.4	233.4	0.023
**Neutrophils (10^9/L)**	4.9	3.7	<0.001	6.0	3.7	<0.001	4.9	3.4	<0.001	4.2	3.6	0.001	4.8	3.6	0.001	3.0	5.9	3.8	0.048	0.087	4.3	3.6	<0.001	3.9	3.8	0.933
**Lymphocytes (10^9/L)**	1.2	1.4	0.009	1.0	1.4	<0.001	1.1	1.5	<0.001	1.4	1.4	0.935	1.1	1.4	<0.001	1.3	1.0	1.4	0.355	0.008	1.3	1.4	0.320	1.4	1.4	0.851
**Monocytes (10^9/L)**	0.55	0.50	0.079	0.53	0.51	0.396	0.57	0.48	<0.001	0.53	0.50	0.029	0.52	0.51	0.601	0.49	0.46	0.51	0.584	0.353	0.56	0.48	<0.001	0.54	0.51	0.170
**Eosinophils (10^9/L)**	0.11	0.09	0.347	0.05	0.10	0.005	0.07	0.11	<0.001	0.11	0.09	0.053	0.08	0.10	0.035	0.07	0.07	0.10	0.174	0.338	0.10	0.09	0.223	0.09	0.10	0.823
**Basophils (10^9/L)**	0.02	0.02	0.902	0.02	0.02	0.581	0.02	0.02	0.922	0.02	0.02	0.652	0.02	0.02	0.098	0.02	0.01	0.02	0.894	0.271	0.02	0.02	0.905	0.02	0.02	0.909
**Neutrophil (%)**	67.8	62.7	<0.001	73.7	62.5	<0.001	69.7	60.8	<0.001	64.9	62.7	0.004	68.0	62.4	<0.001	60.4	71.1	63.4	0.113	0.103	65.3	62.4	<0.001	63.7	63.4	0.821
**Lymphocyte (%)**	21.3	25.9	<0.001	17.1	25.9	<0.001	19.1	27.8	<0.001	23.7	26.0	0.001	21.4	26.1	<0.001	27.8	19.2	25.3	0.117	0.099	23.2	26.4	<0.001	24.8	25.3	0.628
**Monocytes (%)**	8.78	9.32	0.098	7.96	9.35	0.001	9.59	9.10	0.100	9.15	9.29	0.578	9.02	9.29	0.369	9.89	8.19	9.24	0.271	0.405	9.47	9.11	0.179	9.53	9.21	0.392
**Eosinophils (%)**	1.80	1.67	0.655	0.84	1.76	<0.001	1.19	1.90	<0.001	1.79	1.64	0.268	1.28	1.78	0.003	1.45	1.30	1.71	0.441	0.379	1.71	1.68	0.849	1.63	1.70	0.756
**Basophils (%)**	0.38	0.43	0.382	0.32	0.43	0.127	0.40	0.43	0.417	0.39	0.44	0.155	0.34	0.44	0.032	0.47	0.24	0.43	0.644	0.168	0.39	0.44	0.231	0.40	0.43	0.675
**CD3+ T Lymphocytes (%)**	72.2	74.4	0.067	69.9	74.4	0.001	71.5	75.2	<0.001	74.2	74.0	0.856	70.8	74.8	0.001	75.8	72.9	74.0	0.343	0.683	73.9	74.2	0.732	69.3	74.6	<0.001
**CD4+ T Lymphocytes (%)**	44.8	46.6	0.055	43.1	46.6	0.018	45.8	46.5	0.286	46.8	46.1	0.290	44.7	46.6	0.028	48.7	45.5	46.2	0.046	0.773	46.2	46.4	0.818	44.2	46.5	0.044
**CD8+ T Lymphocytes (%)**	24.1	24.3	0.844	24.0	24.3	0.804	22.9	24.9	0.003	24.0	24.4	0.485	23.0	24.6	0.047	23.8	23.9	24.3	0.722	0.870	24.3	24.3	0.984	22.2	24.5	0.023
**CD4+/CD8+ Ratio**	2.2	2.2	0.880	2.3	2.2	0.661	2.4	2.1	<0.001	2.3	2.2	0.246	2.3	2.2	0.243	2.3	2.5	2.2	0.662	0.283	2.2	2.2	0.626	2.3	2.2	0.414
**B Lymphocytes (%)**	14.5	12.4	0.189	14.0	12.4	0.269	12.8	12.6	0.845	13.7	12.2	0.204	14.1	12.4	0.435	7.5	17.7	12.6	0.115	0.488	13.1	12.5	0.542	9.5	13.0	0.049
**NK Cells (%)**	6.0	8.9	0.056	7.0	8.8	0.204	9.1	8.2	0.397	7.6	9.0	0.116	8.8	8.5	0.830	6.8	4.3	8.7	0.586	0.137	8.1	8.7	0.520	11.5	8.2	0.208
**IL-2 (pg/ml)**	3.2	3.0	0.068	3.1	3.0	0.552	2.9	3.1	0.016	3.2	3.0	0.010	3.1	3.0	0.223	2.7	3.5	3.0	0.003	0.122	3.1	3.0	0.053	3.2	3.0	0.046
**IL-4 (pg/ml)**	5.7	2.6	0.287	2.8	3.1	0.834	3.9	2.7	0.413	3.0	3.1	0.845	2.7	3.2	0.662	2.3	3.4	3.1	0.740	0.930	2.9	3.2	0.743	7.5	2.6	0.286
**IL-6 (pg/ml)**	56.2	40.0	0.367	73.4	39.7	0.303	57.5	35.9	0.046	43.2	42.0	0.875	60.5	38.6	0.184	28.7	20.8	43.3	0.460	0.457	42.4	42.3	0.991	43.4	42.3	0.932
**IL-10 (pg/ml)**	4.8	4.1	0.124	5.6	4.1	0.067	4.7	4.0	0.033	4.5	4.1	0.087	5.1	4.0	0.018	3.5	5.4	4.2	0.241	0.236	4.6	4.0	0.008	4.4	4.2	0.676
**TNF-α (pg/ml)**	4.7	5.1	0.510	4.7	5.1	0.640	5.2	5.0	0.784	5.7	4.8	0.066	4.5	5.2	0.207	3.8	3.9	5.1	0.219	0.472	5.6	4.8	0.090	5.7	5.0	0.352
**IFN-γ (pg/ml)**	2.6	2.7	0.812	3.1	2.6	0.490	2.6	2.7	0.858	2.8	2.5	0.063	2.7	2.6	0.948	2.7	3.1	2.6	0.968	0.425	2.6	2.7	0.844	2.8	2.6	0.393
**APTT (s)**	37.8	37.0	0.239	38.9	37.0	0.024	37.9	36.8	0.029	37.5	37.0	0.363	38.6	36.8	0.068	37.4	38.1	37.1	0.786	0.574	37.0	37.2	0.590	36.9	37.2	0.762
**PT (s)**	14.2	13.3	<0.001	14.9	13.4	<0.001	14.1	13.2	<0.001	13.6	13.4	0.195	14.1	13.3	0.002	13.3	14.5	13.5	0.638	0.104	13.7	13.4	0.012	13.6	13.5	0.487
**D-dimer (mg/L)**	2.8	1.3	0.006	5.8	1.1	<0.001	3.1	0.9	<0.001	1.9	1.3	0.059	3.0	1.2	<0.001	0.8	5.4	1.5	0.005	0.074	2.3	1.1	<0.001	1.5	1.5	0.972
**ATIII (%)**	85.6	90.3	0.004	79.4	90.5	<0.001	81.7	93.0	<0.001	89.5	89.6	0.868	85.3	90.5	<0.001	85.9	86.6	89.8	0.077	0.364	88.6	90.2	0.114	86.5	89.9	0.028
**FDP (ug/ml)**	15.9	5.4	0.006	35.0	4.4	<0.001	14.2	3.9	<0.001	8.8	6.0	0.132	14.7	5.3	0.002	3.0	31.1	6.7	0.303	0.129	12.1	3.9	<0.001	7.5	6.9	0.836
**INR**	1.1	1.0	0.001	1.2	1.0	<0.001	1.1	1.0	<0.001	1.1	1.0	0.171	1.1	1.0	0.002	1.0	1.2	1.0	0.648	0.103	1.1	1.0	0.014	1.1	1.0	0.425
**FIB (g/L)**	4.1	4.1	0.626	4.4	4.1	0.164	4.8	3.8	<0.001	4.4	4.0	<0.001	4.8	4.0	<0.001	4.2	4.9	4.1	0.727	0.143	4.5	3.9	<0.001	4.0	4.1	0.400
**TT (s)**	17.4	17.3	0.449	18.2	17.2	0.003	17.9	17.1	<0.001	17.5	17.2	0.006	17.9	17.2	<0.001	17.3	17.2	17.3	0.923	0.739	17.5	17.2	0.007	17.5	17.3	0.369
**Cystatin C (mg/L)**	1.3	1.2	0.132	1.4	1.2	0.007	1.4	1.1	<0.001	1.2	1.2	0.965	1.4	1.1	0.004	1.0	1.7	1.2	0.061	0.189	1.3	1.1	0.001	1.2	1.2	0.271
**LDH (U/L)**	253.2	213.8	0.004	344.3	208.9	<0.001	276.4	195.6	<0.001	253.3	202.9	<0.001	308.6	200.5	<0.001	222.5	394.4	215.8	0.654	0.006	246.9	204.3	<0.001	215.1	219.9	0.638
**Creatine Kinase (U/L)**	121.1	107.6	0.431	185.1	103.2	0.038	117.3	106.3	0.402	150.5	89.6	<0.001	229.1	84.2	<0.001	128.5	87.8	109.2	0.542	0.629	117.3	105.2	0.334	106.7	109.9	0.872
**Urea (mmol/L)**	5.7	4.7	0.024	6.8	4.7	0.004	5.7	4.5	0.001	5.1	4.8	0.225	6.1	4.6	0.005	4.1	7.3	4.9	0.263	0.152	5.3	4.7	0.029	5.0	4.9	0.652
**Creatinine (μmol/L)**	82.5	79.6	0.665	81.9	79.8	0.811	83.8	78.4	0.298	79.2	80.4	0.825	89.8	77.9	0.077	69.0	133.7	79.3	0.340	0.369	88.4	75.3	0.033	82.2	79.7	0.746
**Uric Acid (μmol/L)**	287.9	276.1	0.225	271.8	278.3	0.671	269.2	281.3	0.104	296.4	268.6	<0.001	285.3	276.2	0.363	229.9	269.8	279.9	<0.001	0.748	295.1	268.2	<0.001	266.7	279.1	0.214
**Blood Glucose (mmol/L)**	6.5	6.2	0.271	6.6	6.2	0.333	6.6	6.1	0.010	6.4	6.1	0.110	6.7	6.1	0.016	5.5	6.3	6.3	<0.001	0.906	6.6	6.0	0.001	6.5	6.2	0.373
**Total Chol (mmol/L)**	4.0	4.3	0.003	3.6	4.3	<0.001	3.9	4.4	<0.001	4.3	4.2	0.598	4.0	4.3	<0.001	3.9	3.9	4.3	0.007	0.076	4.3	4.2	0.217	4.3	4.3	0.728
**Triglyceride (mmol/L)**	1.3	1.5	<0.001	1.2	1.5	0.016	1.4	1.6	0.005	1.6	1.4	0.003	1.5	1.5	0.562	1.3	1.5	1.5	0.196	0.870	1.7	1.4	<0.001	1.5	1.5	0.883
**Troponin I (ng/L)**	46.4	13.7	0.231	91.4	12.6	0.135	49.3	5.0	0.040	21.3	17.4	0.785	73.9	6.9	0.076	5.4	263.9	14.5	0.757	0.269	39.0	7.4	0.090	10.1	19.7	0.658
**CK-MB (ng/ml)**	2.0	2.1	0.941	3.3	1.9	0.084	1.9	2.1	0.566	2.3	1.9	0.441	3.3	1.8	0.039	2.6	3.9	2.0	0.543	0.214	1.7	2.2	0.235	1.0	2.2	<0.001
**Blood CO2 (mmol/L)**	23.5	24.1	0.041	23.6	24.0	0.216	23.7	24.1	0.054	24.2	23.9	0.266	23.5	24.1	0.029	22.7	23.6	24.1	0.002	0.702	23.7	24.2	0.025	23.7	24.0	0.338
**LDL Chol(mmol/L)**	2.2	2.3	0.015	2.1	2.3	0.005	2.2	2.3	0.002	2.3	2.3	0.136	2.1	2.3	0.001	2.0	2.0	2.3	0.001	0.100	2.4	2.2	0.004	2.2	2.3	0.486
**HDL Chol (mmol/L)**	1.2	1.3	0.051	1.1	1.3	<0.001	1.1	1.3	<0.001	1.2	1.3	<0.001	1.1	1.3	<0.001	1.2	1.0	1.3	0.636	0.007	1.2	1.3	<0.001	1.4	1.3	0.010

* ULN, Upper Limit of Normal; LLN, Lower Limit of Normal; ALT, Alanine Aminotransferase; AST, Aspartate Aminotransferase; ALP, Alkaline Phosphatase; GGT, Gamma-glutamyl Transferase; LDH, Lactate Dehydrogenase; CK-MB, Creatine Kinase-MB; LDL, Low-density Lipoprotein; HDL, High-density Lipoprotein; WBC, White Blood Cell; RBC, Red Blood Cell; NK, Natural Killer; IL, Interleukin; Chol, Cholesterol.

**Table 3:** Comparison of the unenhanced CT features of multiple organs of adult COVID-19 patients between the abnormal and normal serum hepatic injury markers index groups.

Variables	Total Bilirubin			Direct Bilirubin			Albumin			ALT			AST			ALP					GGT			Total Bile Acid		
	>ULN (n=144)	Normal (n=863)	P	>ULN (n=79)	Normal (n=928)	P	<LLN (n=296)	Normal (n=711)	P	>ULN (n=330)	Normal (n=677)	P	>ULN (n=177)	Normal (n=830)	P	<LLN (n=38)	>ULN (n=20)	Normal (n=949)	P (L)	P (U)	>ULN (n=360)	Normal (n=647)	P	>ULN (n=103)	Normal (n=904)	P
**MCTV of Heart (HU)**	42.4	42.7	0.652	41.3	42.7	0.039	41.8	42.9	0.003	43.3	42.3	0.014	42.1	42.7	0.207	42.1	40.7	42.7	0.554	0.143	42.6	42.6	0.949	42.0	42.7	0.215
**MCTV of Liver (HU)**	55.9	58.0	0.006	54.4	58.0	0.001	56.9	58.0	0.034	55.8	58.7	<0.001	54.6	58.3	<0.001	60.3	54.7	57.7	0.063	0.156	55.2	59.1	<0.001	57.8	57.7	0.904
**< 40 HU**	4	31	0.57	5	30	0.093	7	28	0.001	17	18	0.018	9	26	0.001	1	1	33	0.974	0.817	18	17	<0.001	2	33	0.432
**40-65 HU**	116	659		60	715		246	529		261	514		144	631		30	14	731			297	478		85	690	
**65-70 HU**	15	125		7	133		25	115		35	105		11	129		6	2	132			30	110		10	130	
**>70 HU**	5	28		0	33		4	29		7	26		1	32		1	0	32			3	30		3	30	
**MCTV of Spleen (HU)**	47.1	47.1	0.984	48.5	47.0	0.125	47.0	47.2	0.594	47.6	46.9	0.048	47.1	47.1	0.936	46.2	45.7	47.2	0.268	0.249	46.8	47.3	0.216	46.0	47.2	0.024
**Liver to Spleen Ratio**	1.21	1.24	0.099	1.15	1.25	0.001	1.23	1.24	0.313	1.18	1.27	<0.001	1.17	1.25	<0.001	1.31	1.21	1.24	0.037	0.653	1.19	1.26	<0.001	1.28	1.23	0.074
**< 1.0**	19	90	0.552	16	93	0.004	30	79	0.647	54	55	<0.001	23	86	0.143	1	3	105	0.242	0.659	60	49	<0.001	4	105	0.061
**1.0-1.5**	107	671		53	725		227	551		242	536		130	648		33	12	733			262	516		85	693	
**> 1.5**	12	81		3	90		23	70		23	70		10	83		4	2	87			23	70		10	83	
**MCTV of Abd Aorta(HU)**	40.4	39.9	0.356	40.8	39.9	0.242	39.1	40.4	0.003	40.0	40.0	0.952	39.9	40.0	0.819	40.2	38.5	40.0	0.892	0.295	39.9	40.1	0.576	39.1	40.1	0.110
**MCTV of Pancreas (HU)**	38.3	39.3	0.173	36.8	39.4	0.010	37.1	40.0	<0.001	39.0	39.3	0.678	37.6	39.5	0.008	40.4	41.0	39.1	0.367	0.369	38.4	39.6	0.026	38.2	39.3	0.220
**MCTV of L-Kidney(HU)**	28.4	28.9	0.191	28.3	28.8	0.437	28.2	29.0	0.008	28.8	28.8	0.794	28.3	28.9	0.097	29.7	27.5	28.8	0.164	0.231	28.6	28.9	0.174	28.7	28.8	0.747
**MCTV of R-Kidney(HU)**	29.1	29.6	0.202	28.8	29.6	0.121	29.1	29.7	0.036	29.6	29.5	0.474	29.1	29.6	0.155	30.2	28.6	29.5	0.256	0.402	29.4	29.6	0.489	29.2	29.6	0.331
**Ant Tho Diameter (cm)**	22.0	21.9	0.426	22.2	21.9	0.259	22.2	21.8	0.008	22.5	21.6	<0.001	22.3	21.8	0.009	20.8	21.5	22.0	<0.001	0.337	22.6	21.6	<0.001	22.1	21.9	0.469
**Tra Tho Diameter (cm)**	30.6	31.0	0.153	30.7	30.9	0.407	30.4	31.1	<0.001	31.3	30.7	0.001	31.0	30.9	0.641	30.4	30.3	30.9	0.241	0.325	31.4	30.6	<0.001	30.5	31.0	0.104
**Ant/Tra (Tho)**	0.72	0.71	0.030	0.73	0.71	0.041	0.73	0.70	<0.001	0.72	0.71	<0.001	0.72	0.71	0.028	0.68	0.71	0.71	0.010	0.849	0.72	0.71	0.001	0.73	0.71	0.010
**Ant+Tra (Tho) (cm)**	52.6	52.8	0.660	52.8	52.8	0.939	52.5	52.9	0.165	53.8	52.3	<0.001	53.1	52.7	0.266	50.5	51.8	52.9	<0.001	0.262	53.9	52.2	<0.001	52.5	52.8	0.539
**Ant Abd Diameter (cm)**	22.5	22.3	0.637	22.8	22.3	0.165	22.5	22.3	0.389	23.5	21.8	<0.001	23.1	22.2	<0.001	20.7	21.8	22.4	<0.001	0.396	23.6	21.7	<0.001	22.5	22.3	0.589
**Tra Abd Diameter (cm)**	29.9	29.7	0.593	30.2	29.7	0.128	30.0	29.7	0.080	30.6	29.4	<0.001	30.4	29.6	0.001	28.7	30.2	29.8	0.014	0.558	30.7	29.2	<0.001	29.7	29.8	0.809
**Ant/Tra (Abd)**	0.75	0.75	0.828	0.75	0.75	0.567	0.75	0.75	0.577	0.77	0.74	<0.001	0.76	0.75	0.018	0.72	0.72	0.75	0.001	0.032	0.77	0.74	<0.001	0.76	0.75	0.187
**Ant+Tra (Abd) (cm)**	52.3	52.1	0.600	53.0	52.1	0.129	52.5	52.0	0.180	54.1	51.2	<0.001	53.6	51.8	<0.001	49.4	52.0	52.2	0.001	0.859	54.3	50.9	<0.001	52.2	52.1	0.857
**Tho+Abd (Ant+Tra) (cm)**	105.0	104.9	0.918	105.9	104.8	0.360	105.0	104.9	0.889	107.9	103.5	<0.001	106.7	104.6	0.007	99.9	103.8	105.2	0.001	0.542	108.2	103.1	<0.001	104.8	104.9	0.859
**Abd/Tho (Ant+Tra)**	0.99	0.99	0.236	1.00	0.99	0.026	1.00	0.98	<0.001	1.01	0.98	<0.001	1.01	0.98	<0.001	1.00	1.00	0.99	0.770	0.160	1.01	0.98	<0.001	0.99	0.99	0.329
**Fat Thickness (mm)**	15.4	17.3	0.002	13.6	17.3	<0.001	14.7	17.9	<0.001	16.7	17.1	0.322	15.9	17.2	0.020	16.8	12.3	17.1	0.819	0.003	16.5	17.3	0.082	16.9	17.0	0.863
Fat Thickness*10/ **Tho Diameter (Ant+Tra)**	0.29	0.32	0.001	0.25	0.32	<0.001	0.28	0.34	<0.001	0.31	0.32	0.034	0.30	0.32	0.005	0.33	0.24	0.32	0.546	0.003	0.30	0.33	0.001	0.32	0.32	0.893
Fat Thickness*10/ **Abd Diameter (Ant+Tra)**	0.29	0.33	<0.001	0.25	0.33	<0.001	0.28	0.34	<0.001	0.31	0.33	0.002	0.30	0.33	<0.001	0.34	0.24	0.33	0.546	0.003	0.30	0.34	<0.001	0.32	0.33	0.768
Fat Thickness*10/ **Tho+Abd (Ant+Tra)**	0.15	0.16	0.001	0.13	0.16	<0.001	0.14	0.17	<0.001	0.15	0.16	0.008	0.15	0.16	0.002	0.17	0.12	0.16	0.576	0.003	0.15	0.17	<0.001	0.16	0.16	0.832

* ULN, Upper Limit of Normal; LLN, Lower Limit of Normal; ALT, Alanine Aminotransferase; AST, Aspartate Aminotransferase; ALP, Alkaline Phosphatase; GGT, Gamma-glutamyl Transferase; MCTV, Mean CT Value; Abd, Abdominal; Tho, Thoracic; Ant, Anteroposterior; Tra, Transverse.

# Fat Thickness represents abdominal subcutaneous fat thickness (Measured on the slice of the superior mesenteric artery originated from the abdominal aorta and at the midline of the abdomen).

Seventy-nine patients (7.85%) had elevated serum direct bilirubin (DBil). Males had a higher proportion (13.3%) of elevated DBil than females (5.0%). The mean age of patients with abnormal DBil was 64.0 y, which was greater than that of patients with normal DBil *(P = 0.011)*. Although univariate analysis showed that there were differences in 26 blood indicators between the two groups of patients (elevated vs. normal DBil) (**Table 2**), multivariate analysis indicated that only three serum markers (lactate dehydrogenase, LDH; total cholesterol; and low-density lipoprotein cholesterol) were independently related to abnormal DBil (**Table 1**). The liver MCTV, the liver MCTV/spleen MCTV ratio, the subcutaneous fat thickness and the ratio of subcutaneous fat thickness to the sum of the transverse and anteroposterior abdominal diameters were significantly reduced (**Table 3**). **Table 1** indicates that females were less likely to develop elevated DBil than males (OR: 0.36, 95% CI: 0.16-0.83). Adult COVID-19 patients who had a history of HBV infection had an elevated risk of abnormal DBil (OR: 3.26, 95% CI: 1.22-8.69).

There were 296 patients (29.4%) with decreased serum albumin. Albumin was below the lower limit of the normal range in more than one-third of males but in only approximately one-fifth of females. The mean age of the patients with reduced albumin was more than ten years older than that of the patients with normal albumin *(P<0.001)*. The incidence of chronic diseases (such as hypertension and diabetes) was increased among COVID-19 patients with decreased serum albumin. Binary logistic regression analysis showed that twenty indicators were independently associated with reduced serum albumin in adult COVID-19 patients (**Table 1**).

Three hundred thirty patients (32.8%) had elevated serum ALT. One hundred and seventy-seven patients (17.6%) had elevated serum AST. The proportions of males were greater than the proportions of females in both the elevated ALT group and the elevated AST group, with P values less than 0.001. The patients with abnormal ALT were younger (mean age: 58.0 y) than the patients with normal ALT (mean age: 60.9 y), with a *P* value of 0.004. However, the mean age of the abnormal AST group (63.7 y) was greater than the mean age of the normal AST group (59.2 y), with a *P* value less than 0.001. Univariate analysis showed that the blood indexes and CT scan indicators that differed between the abnormal ALT and normal ALT groups were not the same as those that differed between the abnormal AST and normal AST groups (**Tables 2**, **3**). The factors simultaneously independently associated with abnormal ALT and AST were sex (OR: 0.20 and 0.42), age (OR: 0.98 and 1.02), the ratio of the sum of the abdominal diameters to the sum of the thoracic diameters (OR: 59.6 and 522.2), and the ratio of abdominal fat thickness to the sum of the abdominal diameters (OR: 0.12 and 0.15). Other indicators independently correlated with increased ALT or AST in adult COVID-19 patients are shown in **Table 1**.

Among all patients, only a small number had abnormal serum ALP (38 had decreased ALP, and 20 had increased ALP). Compared to the patients with normal ALP, the patients with low ALP were significantly younger *(P = 0.008)*; although patients with elevated ALP were also younger, the difference was not significant *(P = 0.733)*. In binary logistic regression analysis, there were few independent factors related to abnormal ALP (**Table 1**), perhaps because of the small number of abnormal ALP cases in our study or the lack of inclusion of other relevant factors.

Three hundred and sixty patients (35.8%) had elevated serum GGT. The proportion of males with abnormal GGT was significantly higher than that of females *(P<0.001)*. Among adult COVID-19 patients, more patients with elevated GGT had chronic diseases (hypertension or type 2 diabetes) and unhealthy lifestyles (overweight, smoking or drinking) than patients with normal GGT, with all *P* values less than 0.05. Independent-sample t-test analysis revealed that 23 blood indexes were significantly different between the abnormal and normal GGT groups. As shown in **Table 1**, TNF-α, FDP, FIB, LDH, uric acid and triglycerides in blood were positively associated with elevated serum GGT. In addition, four CT scan indicators were independently associated with elevated serum GGT, including the liver MCTV (OR: 0.97, 95% CI: 0.95-0.99), the anteroposterior abdominal diameter (OR: 0.82, 95% CI: 0.72-0.94), the anteroposterior abdominal diameter (OR: 1.39, 95% CI: 1.26-1.53) and the ratio of abdominal subcutaneous fat thickness to the sum of the abdominal anteroposterior and transverse diameters (OR: 0.04, 95% CI: 0.01-0.14).

There were 103 patients (10.2%) with elevated serum TBA. Patients with elevated TBA were older *(P<0.001)* and had a higher proportion of type 2 diabetes *(P = 0.01)* than those without elevated TBA. The mean values of blood indicators such as red blood cells (RBCs), platelets, CD3+ T lymphocytes, CD4+ T lymphocytes, CD8+ T lymphocytes, B lymphocytes, IL-2, ATIII, CK-MB, and HDL cholesterol in the abnormal TBA group were different from those in the normal TBA group, but multivariate analysis showed no significant differences. Type 2 diabetes (OR: 2.78, 95% CI: 1.30-5.92), a ratio of liver MCTV to spleen MCTV less than 1.0 (OR: 0.24, 95% CI: 0.07-0.77) and a ratio of anteroposterior diameter to transverse diameter of the chest were independently correlated with elevated serum TBA (**Table 1**).

## 4 Discussion

Our study showed that approximately 69.2% of all adult COVID-19 patients had at least one abnormal serum hepatic injury markers marker. Univariate analysis and binary logistic regression analysis showed that the factors associated with each of the eight abnormal hepatic injury markers differed. However, our results suggested that the factors independently related to abnormalities in most of the eight hepatic injury markers indexes were sex, age, a history of HBV infection, blood FIB and LDH, the liver MCTV, the pancreas MCTV, and the ratio of abdominal subcutaneous fat thickness to the abdominal diameters.

We found that females accounted for a larger proportion of adult COVID-19 patients than males. However, the proportion of male patients with abnormal hepatic injury markers indicators was higher than that of female patients with abnormal hepatic injury markers indicators. Our results showed that sex was independently associated with abnormalities in six of the eight hepatic injury markers (TBil, DBil, albumin, ALT, AST, and GGT) and that adult male COVID-19 patients were more likely to have abnormal hepatic injury markers than female patients. Other studies have similarly shown that among patients with other etiologies that cause hepatocyte damage (such as HBV infection and non-alcoholic liver disease), the proportion of males with abnormal hepatic injury markers is greater than the proportion of females with abnormal hepatic injury markers (Xiao et al., 2015; Xiao et al., 2017; Tobari and Hashimoto, 2020). We speculate that this result may be due in part to a more unhealthy lifestyle (smoking, alcohol drinking, etc.) among men than among women (Takenaka et al., 2020; Kezer et al., 2021). In addition, many experimental studies have indicated that the sex-biased hepatic expression of genes is correlated with sex differences in the incidence and progression of many liver diseases (Zhang et al., 2012; Luo et al., 2019; Lau-Corona et al., 2020).

Li’s research showed that COVID-19-induced hepatic injury markers abnormalities are associated with age (Li et al., 2020). Our results indicated that COVID-19 patients with elevated ALT or decreased ALP tended to be younger, while patients with elevated AST or reduced albumin tended to be older. Other studies have demonstrated that age-related chronic inflammation can promote hepatocyte senescence and chronic liver disease (Gómez-Santos et al., 2020; Bianchi et al., 2021). This mechanism may explain why age is related to abnormal hepatic injury markers to a certain extent. Ma and colleagues showed that hepatic deoxycholic acid levels vary with age in mice (Ma et al., 2020). Although the patients with abnormal TBil, DBil, GGT or TBA tended to be older, age was not an independent factor associated with abnormalities in these four hepatic injury markers.

Our results showed that adult COVID-19 patients with a history of HBV infection had elevated risks of increased DBil and AST. However, HBV infection history was not independently correlated with abnormalities in the other six hepatic injury markers indicators. Lin and colleagues revealed that patients with inactive HBV and SARS-CoV-2 coinfection are at an increased risk of abnormal hepatic injury markers (Lin et al., 2020). In our study, 19 of 129 patients with HBV infection history were positive for hepatitis B surface antigen (HBsAg), while the others were positive only for hepatitis B core antibody (HBcAb) or both HBcAb and HBeAb. Previous research has shown that some people who are positive only for HBcAb may test positive for HBV DNA (El-Nabi et al., 2020; Khiangte et al., 2020). Because few people were tested for HBV DNA in this study, the number of patients positive only for HBcAb who were in the active stage of HBV infection is unknown.

The univariate analysis showed that the mean values of multiple blood variables differed between the abnormal and normal hepatic injury markers index groups. However, binary regression analysis indicated that the main factors related to most of the abnormal serum hepatic injury markers indexes were FIB and LDH. Effenberger suggested that COVID-19 patients with elevated AST exhibit significantly elevated LDH and that systemic inflammation could drive hepatic injury in COVID-19 patients (Effenberger et al., 2021). Tsutsumi and colleagues proposed that an elevated D-dimer level is independently associated with ALT elevation and that liver dysfunction in COVID-19 patients might be induced by microvascular thrombosis (Tsutsumi et al., 2021). However, in a mouse model, intravascular fibrinogen was measured following acute liver injury (Poole et al., 2021).

We found that the MCTVs of the liver and pancreas were independently related to most of the abnormal serum hepatic injury markers in adult COVID-19 patients. The liver density and pancreas density were reduced (with reduced MCTVs of the liver and panaceas) in adult COVID-19 patients with abnormal hepatic injury markers in our study. Many other clinical or experimental studies have confirmed the correlation between fat deposition in the liver and fat deposition in the pancreas (Cohen et al., 2018; Taylor et al., 2018; Aliyari Ghasabeh et al., 2020; Chiyanika et al., 2020). It is unclear whether the liver dysfunction in COVID-19 is due to acute diffuse liver injury caused by SARS-CoV-2 or to acute diffuse liver injury caused by previous fatty infiltration. Research has shown that abdominal subcutaneous fat thickness is a reliable indicator of the severity of liver disease in obese nondiabetic individuals (Hegazy et al., 2019). Our study showed that although the subcutaneous fat thickness was reduced in adult COVID-19 patients with abnormal hepatic injury markers indexes, it was not an independent risk factor for abnormal hepatic injury markers indicators. This result may be attributable to the fact that few patients were obese in our study. Notably, researchers have shown that the mean body mass index of Chinese people is much lower than that of people in the Western world (Pang et al., 2020). However, our results demonstrated that the ratio of subcutaneous fat thickness to the sum of the transverse and anteroposterior abdominal diameters on the unenhanced CT scan was independently associated with most of the abnormal serum hepatic injury markers.

There were some limitations in this study. First, this was a retrospective study from a single center with a moderate number of subjects. Second, a portion of the included patients had a history of HBV infection, but very few patients tested positive for HBV DNA in the blood. In addition, the anti-HBV treatments were unknown. The results may be different from those of studies that have included patients with hepatitis C virus infection (Krist et al., 2020; Owens et al., 2020). Third, it was unclear whether any of the included patients had a history of alcoholic or non-alcoholic fatty liver disease. Fourth, due to the immune response caused by SARS-CoV-2 infection, immunoglobulin was produced in most patients, resulting in serum total protein levels in the normal range in most patients. Therefore, we did not specifically analyze the characteristics of total protein and the ratio of albumin to globulin. Fifth, because most of the patients did not undergo scanning of the lower abdomen on CT examination, we measured the subcutaneous fat thickness and abdominal transverse and anteroposterior diameters in the plane of the superior mesenteric artery originating from the abdominal aorta using PACS software. Sixth, we did not record the patients’ abdominal circumference data, and it is difficult to measure this variable with PACS software. The sum of the abdominal transverse and anteroposterior diameters may not adequately represent the abdominal circumference. Seventh, Given the observational nature of our study, the proposed relationships between abdominal fat distribution, organ density, and hepatic injury markers should be interpreted as associative rather than causal. Future prospective imaging–pathology correlation studies and mechanistic investigations are warranted to validate these hypotheses and clarify the biological pathways involved. Finally, the drugs and duration of anti-SARS-CoV-2 treatments before admission were not detailed. Antivirotic drugs may cause damage to human hepatocytes, leading to abnormal serum hepatic injury markers; this possibility needs to be studied further.

## 5 Conclusions

In conclusion, sex, age, HBV infection, serum FIB content, blood LDH concentration, liver and pancreas MCTVs and the ratio of abdominal subcutaneous fat thickness to the sum of the transverse and anteroposterior abdominal diameters were independently correlated with abnormalities in many serum hepatic injury markers in adult COVID-19 patients. We look forward to prospective multicenter studies and experimental studies that will confirm the results.

## Data Availability

The original contributions presented in the study are included in the article/**Supplementary Material**. Further inquiries can be directed to the corresponding author.

## Supplementary material

Supplementary Figure 1 We measured the abdominal transverse and anteroposterior diameters in the plane of the superior mesenteric artery **(B)** originating from the abdominal aorta **(A)** for each individual using PACS software.
